# Is the relationship between the built environment and physical activity moderated by perceptions of crime and safety?

**DOI:** 10.1186/1479-5868-11-24

**Published:** 2014-02-24

**Authors:** Nicole L Bracy, Rachel A Millstein, Jordan A Carlson, Terry L Conway, James F Sallis, Brian E Saelens, Jacqueline Kerr, Kelli L Cain, Lawrence D Frank, Abby C King

**Affiliations:** 1 Harder + Company Community Research and San Diego State University, School of Public Affairs, 3965 Fifth Avenue, Suite 420, San Diego, CA 92103, USA; 2 SDSU/UCSD Joint Doctoral Program in Clinical Psychology, 3900 Fifth Avenue, Suite 310, San Diego, CA 92103, USA; 3 Department of Family and Preventive Medicine, University of California, San Diego, 3900 Fifth Avenue, Suite 310, San Diego, CA 92103, USA; 4 Department of Pediatrics, University of Washington & Seattle Children’s Research Institute, 2001 Eighth Avenue, Suite 400, Seattle, WA 98121, USA; 5 School of Community and Regional Planning, University of British Columbia, Vancouver BC, #433-6333 Memorial Road, Vancouver BC V6T 1Z2, Canada; 6 Department of Health Research & Policy and Stanford Prevention Research Center, Department of Medicine, Stanford University School of Medicine, 259 Campus Drive, HRP Redwood Building, T221, Stanford, CA 94305-5405, USA

**Keywords:** Ecological models, Older adults, Parks, Social environment, Walkability, Traffic, Transportation

## Abstract

**Background:**

Direct relationships between safety concerns and physical activity have been inconsistently patterned in the literature. To tease out these relationships, crime, pedestrian, and traffic safety were examined as moderators of built environment associations with physical activity.

**Methods:**

Exploratory analyses used two cross-sectional studies of 2068 adults ages 20–65 and 718 seniors ages 66+ with similar designs and measures. The studies were conducted in the Baltimore, Maryland-Washington, DC and Seattle-King County, Washington regions during 2001–2005 (adults) and 2005–2008 (seniors). Participants were recruited from areas selected to sample high- and low- income and walkability. Independent variables perceived crime, traffic, and pedestrian safety were measured using scales from validated instruments. A GIS-based walkability index was calculated for a street-network buffer around each participant’s home address. Outcomes were total physical activity measured using accelerometers and transportation and leisure walking measured with validated self-reports (IPAQ-long). Mixed effects regression models were conducted separately for each sample.

**Results:**

Of 36 interactions evaluated across both studies, only 5 were significant (*p* < .05). Significant interactions did not consistently support a pattern of highest physical activity when safety was rated high and environments were favorable. There was not consistent evidence that safety concerns reduced the beneficial effects of favorable environments on physical activity. Only pedestrian safety showed evidence of a consistent main effect with physical activity outcomes, possibly because pedestrian safety items (e.g., crosswalks, sidewalks) were not as subjective as those on the crime and traffic safety scales.

**Conclusions:**

Clear relationships between crime, pedestrian, and traffic safety with physical activity levels remain elusive. The development of more precise safety variables and the use of neighborhood-specific physical activity outcomes may help to elucidate these relationships.

## Background

Physical activity is important for the prevention of chronic diseases and promotion of health, yet most Americans do not meet national guidelines [1]. A comprehensive understanding of the correlates of physical activity can inform the development of effective interventions. Prior research documented a variety of factors related to physical activity in both younger adults and older adults, including built environment features such as neighborhood walkability and proximity of parks and recreation facilities [2-4], and neighborhood social environment factors such as aesthetics and incivilities [5-7]. Given that barriers to physical activity exist on multiple levels – individual, social, and environmental – employing an ecological model to guide research on these multiple levels of influence is useful. An important principle of ecological models is that interactions can occur across levels, such as with built environment and social environment factors, e.g., crime [8].

Crime, traffic, and pedestrian-related safety are neighborhood environment variables that may affect people’s willingness to be physically active outdoors in their neighborhoods [9-11]. Crime and traffic safety are social environment variables, but pedestrian safety, as measured here, mainly deals with perceptions of pedestrian infrastructure in the built environment, including street crossing aids. However, the evidence demonstrating the relations between these constructs and physical activity has been inconsistent [5]. In a review of 18 studies examining environmental correlates of walking, traffic and pedestrian-related safety were positively associated with walking for exercise, recreation and transportation, but not associated with total walking in adults [7]. The relation between crime-related safety and physical activity is even less clear. Most studies of adults, with some exceptions, have not found significant associations between crime-related safety and physical activity [12].

Studies assessing perceived crime safety and physical activity specifically in older adults have also produced mixed results [13]. However, several found inverse associations between perceptions of danger and less physical activity among older adults [14-16]. It is reasonable to expect that older adults’ outdoor physical activity is more sensitive to their perceptions of safety, as they are generally more physically vulnerable, unable to move quickly out of the path of an oncoming car or defend themselves against a mugger on the street, for example. Therefore, it is useful to examine older adults as a distinct group (from their younger counterparts) in assessing how safety perceptions interact with built environment factors to explain physical activity.

The mixed results found in the literature suggest that the relations of crime, traffic, and pedestrian safety with physical activity may be more complex than through direct associations. It is possible, for example, that perceived crime safety and concern for pedestrian and traffic safety may have interactive effects with built environment constructs rather than main effects on physical activity.

The present study tested hypotheses based on an ecological model, that the association of the built environment and physical activity might be moderated by perceptions of neighborhood crime, traffic, and pedestrian safety. Based in part on the literature [4,7], walkability was expected to interact with crime, traffic and pedestrian safety variables in explaining walking for transportation and total physical activity. The number of proximal parks and recreation facilities was expected to interact with the crime, traffic and pedestrian safety variables in explaining walking for leisure and total physical activity. If, for example, participants feel safe walking in their neighborhoods and have one or more parks or recreation facilities nearby, they may be more likely to walk, particularly for leisure. Generally, built environment attributes were expected to have stronger associations with physical activity when perceived safety was high as opposed to when perceived safety was low. Walking for transportation was expected to be less-related to safety variables for some groups, such as those with no or limited access to a private vehicle, which would be more common among low-income subgroups. Thus, we anticipated lower associations of perceived safety with walking for transportation than with walking for leisure.

Separate samples of younger and older adults made possible a richer test of hypotheses and an informal examination of whether safety perceptions appear to moderate the built environment-physical activity relationship differently for these groups. Both samples were recruited from the same two distinct regions of the US, using a systematic sampling approach designed to produce a sample with wide variability in neighborhood built and socioeconomic environments. The physical vulnerability of older adults led to the expectation that moderating effects of safety would be stronger for older adults than younger adults. Sample-specific, instead of combined, analyses were used due to some differences in methods across samples.

## Methods

### Design

Present analyses used data from separate studies of younger adults and older adults that were similar in their design and methods. Both studies were approved by the Institutional Review Board at San Diego State University, and informed written consent was obtained from all participants.

#### NQLS

The Neighborhood Quality of Life Study (NQLS) was an observational epidemiologic study designed to examine relations among built environment and income variables, physical activity, and other health outcomes among adults aged 20–65. NQLS was conducted in the Baltimore, Maryland-Washington, DC and Seattle-King County, Washington metropolitan areas during 2001–2005. Neighborhoods were defined as clusters of contiguous census block groups that met eligibility criteria for median household income and walkability. Sixteen neighborhoods were included from each metro area, evenly divided by walkability (high/low) and income (high/low). Details of neighborhood selection, walkability index calculations, and results have been previously reported [17,18].

#### SNQLS

The Senior Neighborhood Quality of Life Study (SNQLS) was based on a similar stratified design and was conducted in the same regions during 2005–2008. Important differences from the NQLS study were that the SNQLS sample was aged 66 and older, and participants were recruited from individual census block groups (N = 216) that met criteria for high and low walkability and high and low income, instead of contiguous clusters of block groups. SNQLS study design details and initial results are detailed elsewhere [19].

### Participants and procedures

#### NQLS

Participants were 2199 adults recruited from households in the identified neighborhoods, using marketing company mailing lists, and contacted by phone and mail. Eligibility criteria included: being 20–65 years old, residing in a private home (not a group facility), proficiency in speaking and reading English, and the ability to walk independently. Participants signed a consent form and then were mailed an accelerometer. Accelerometers were worn for one week, and participants completed the survey at the end of that week. Surveys could be completed by mail, online, or telephone interview. Participants received $20 for completing the survey and returning the accelerometer [18].

#### SNQLS

Participants were 718 adults age 66 or greater who lived independently in the community (not in a group facility). Participants were identified and recruited using similar methods as NQLS. Eligibility criteria for SNQLS were similar to NQLS, with the addition of: being able to correctly reiterate the study tasks and reporting ability to walk more than 10 feet (approximately 3 meters) at a time. The data collection method was the same as described above. Incentive payments of $25 were given to participants who returned accelerometer and survey data [19].

### Measures

Measures were generally similar across studies, and most differences were due to ensuring that measures were appropriate for each of the age groups.

#### Demographics

For both studies, age, gender, ethnicity (non-Hispanic white vs. other), education (5 levels from ‘less than high school’ to ‘graduate degree’), number of motor vehicles per adult in household, number of people in household, years at current address, and marital status (re-categorized as married/living together or other) were collected by survey.

#### Perceived safety measures

Perceived, rather than objective, measures of safety were used for two key reasons: first, the lack of availability of comparable crime data across jurisdictions and lack of objective data on traffic and pedestrian safety variables; and second, participants’ perceptions of safety were thought to be more likely to be related to outdoor physical activity behaviors. For example, a neighborhood may have a high crime rate, as measured by police indicators, but if a resident of that neighborhood does not perceive threats, the crime rate may have no bearing on physical activity. Both studies used modified scales from the Neighborhood Environment Walkability Scale (NEWS) to assess perceptions of traffic, pedestrian, and crime safety. Reliability and validity of the NEWS have been supported by multiple studies [20-22]. Traffic safety was measured using 5-item (NQLS α = 0.6) and 3-item (SNQLS α = 0.6) scales asking participants to rate their perception of the quantity and speed of traffic on their neighborhood streets. The NQLS survey included 2 additional items in this section asking specifically about the quantity and speed of traffic on the street on which the respondent lives (as opposed to SNQLS which asks about “nearby” streets only). Pedestrian safety was measured using 7-item (NQLS α = 0.7) and 9-item (SNQLS α = 0.5) scales asking participants to rate their perception of how safe it is to walk in their neighborhoods, specific to issues such as safe intersections, sidewalks and crosswalks. The SNQLS survey included additional items in this section that were more relevant to seniors’ pedestrian safety, such as whether the crosswalks in their neighborhood were designed for people who do not see well. Crime safety was measured using 4-item (NQLS α = 0.7) and 5-item scales (SNQLS α = 0.8) addressing perceptions of neighborhood crime. The SNQLS crime safety scale included one additional item thought to be particularly relevant to older adults’ safety concerns: teenagers hanging out in the neighborhood. Higher scores on each scale corresponded to better safety. Table 1 shows the details of the safety items completed by each sample.

**Table 1:** Safety items used in the present study

**Adult sample**	**Older adult sample**
Crime safety	
1. There is a high crime rate in my neighborhood.	1. There is a high crime rate in my neighborhood.
2. The crime rate in my neighborhood makes it unsafe to go on walks during the day.	2. The crime rate in my neighborhood makes it unsafe to go on walks during the day.
3. The crime rate in my neighborhood makes it unsafe to go on walks at night.	3. The crime rate in my neighborhood makes it unsafe to go on walks at night.
4. My neighborhood is safe enough so that I would let a 10-year-old boy walk around my block alone in the daytime^a^.	4. There are alleys between buildings that make it unsafe to walk in my neighborhood^a^.
	5. There are teenagers hanging out that make it unsafe to walk in my neighborhood^a^.
Pedestrian safety	
1. My neighborhood streets are well lit at night.	1. My neighborhood streets are well lit at night.
2. Walkers and bikers on the streets in my neighborhood can be easily seen by people in their homes.	2. Walkers and bikers on the streets in my neighborhood can be easily seen by people in their homes.
3. There are unattended or stray dogs in my neighborhood.	3. Stray or loose dogs can be a problem in my neighborhood.
4. There are crosswalks and pedestrian signals to help walkers cross busy streets in my neighborhood.	4. Pedestrian signals in my neighborhood give me enough time to cross the road^a^.
5. The crosswalks in my neighborhood help walkers feel safe crossing busy streets.	5. The crosswalks in my neighborhood are designed for people who don’t see well because they have things like beeps that tell you when to cross.^a^
6. When walking in my neighborhood there are a lot of exhaust fumes (such as from cars, buses).	6. At major intersections in my neighborhood, there are islands in the middle of the road where pedestrians can safely stop after crossing half way^a^.
7. I see and speak to other people when I am walking in my neighborhood.	7. I have to cross many busy streets to get to places like shops in my neighborhood^a^.
	8. Cars going across sidewalks to get to driveways and parking lots make it difficult to walk in my neighborhood^a^.
	9. There are curb cuts (ramps) that go from sidewalk level to road level in my neighborhood^a^.
Traffic safety	
1. There is so much traffic along the street I live on that it makes it difficult or unpleasant to walk in my neighborhood.	1. There is so much traffic along nearby streets that it makes it difficult or unpleasant to walk in my neighborhood.
2. There is so much traffic along nearby streets that it makes it difficult or unpleasant to walk in my neighborhood.	2. The speed of traffic on most nearby streets is usually slow (30 mph or less).
3. Most drivers exceed the posted speed limits while driving in my neighborhood.	3. Most drivers exceed the posted speed limits while driving in my neighborhood.
4. The speed of traffic on the street I live on is usually slow (30 mph or less).	
5. The speed of traffic on most nearby streets is usually slow (30 mph or less).	

^a^Derived for present study.

Note: All items were scored on a 4-point scale ranging from strongly disagree to strongly agree; scales were scored so that higher numbers represented greater perceived safety.

#### Objective built environment measures

Data from the county-level tax assessor, regional land use at the parcel level, and street networks were integrated into geographic information systems (GIS) to create a walkability index for each participant based on a 1000 meter (NQLS) and 500 meter (SNQLS) street-network buffer around his/her home. The index consisted of the sum of z-scores of measures of residential density, retail floor area ratio, intersection density, and land use mix [17]. The different buffer sizes were chosen because younger adults were expected to walk further than older adults. For the SNQLS sample, measures using both buffer sizes were analyzed, and the 500 meter buffer produced consistently stronger findings; therefore, only results using the 500 meter buffer measures are presented in this paper.

Parcel-level land use data, supplemented with lists from local parks agencies, were used to determine the number of parks within or intersecting the 1000 (NQLS) or 500 (SNQLS) meter buffer around each participant’s home. Using paper and internet-based phone directories, private recreation facilities (e.g., gyms, dance and martial arts studios) within each region were identified and geocoded, as described elsewhere [23]. The count of parks and private recreation facilities within 1000 (NQLS) or 500 (SNQLS) meters of each participant’s home was calculated separately and dichotomized as 0 or ≥ 1.

#### Physical activity

For both studies, ActiGraph accelerometers (Manufacturing Technology Incorporated, models 7164 and 71256; Pensacola, FL) were used to objectively measure participants’ total physical activity. Accelerometers have been validated for adults [24] and older adults [25]. The epoch was set at 60-seconds and participants were required to wear the accelerometer for at least 5 valid days. Eight hours of valid wear time was required for a valid day, and a valid hour contained no more than 30 (NQLS) or 45 (SNQLS) consecutive minutes of zero counts. Data were cleaned and scored using MeterPlus version 4.0 software from Santech, Inc. (http://www.meterplussoftware.com). Cumulative minutes/week (as opposed to continuous bouts) of moderate to vigorous physical activity (MVPA) was calculated for both samples using previously established cut-points for adults (≥1952 counts/minute) [26].

For NQLS, self-reported physical activity was assessed using the well-validated International Physical Activity Questionnaire (IPAQ) survey [27]. Items assessed frequency and duration of transportation and leisure walking within the past week, from which average minutes/week of the two domains of walking were calculated.

For SNQLS, self-reported physical activity was assessed using the Community Healthy Activities Model Program for Seniors (CHAMPS) survey. Six-month stability was acceptable (ICCs = 0.58-0.67), and the measure was able to discriminate between inactive, somewhat active, and active persons [28]. An average minutes/week variable was computed for the walking for transportation single item: “In a typical week during the past 4 weeks did you walk *to do errands* (such as to/from a store or to take children to school)?” and for the walking for leisure single item: “In a typical week during the past 4 weeks did you walk *leisurely* for exercise or pleasure?” Those reporting any walking indicated categories of minutes/week.

### Analysis

Eighteen mixed effects regression models were conducted for NQLS and SNQLS samples separately using SPSS version 17.0 with census block group entered as a random effect variable to account for clustering, and accelerometer-derived total MVPA, walking for transportation, and walking for leisure entered as dependent variables. The intraclass correlation coefficient (ICC) assessing proportion of variance between block groups was examined for each outcome variable with no other variables in the model. Models were created using conceptually matched variables based on ecological models [29,30] and previous research. For example, walkability was not expected to be related to walking for leisure, and parks and recreation facilities were not expected to be related to walking for transportation. Separate models were fitted to test each interaction term (18 models in each sample) to maximize sensitivity for detecting interaction effects and reduce chance for type 2 error. Continuous independent variables were grand mean centered, and dichotomous variables were centered on 0. The two NQLS IPAQ walking outcomes (transportation and leisure) were skewed (skewness > 2.0), so the natural log of those variables was used (after adding a constant of 1 to all cases to remove the problem of a value of 0) in the regression models. Unstandardized coefficients (B) with 95% confidence intervals (CI) were reported to represent change in (log of) minutes/week of physical activity for every 1 unit change in continuous predictors or presence/absence of parks and recreation facilities (i.e., dichotomous predictors). The eight demographic variables mentioned in the measures section were controlled for in each model. Pseudo R^2^ for neighborhood-level variance (i.e., neighborhood-level variance explained by the built environment and safety variables and their interaction) was calculated for models with significant interactions by taking the (intercept from null model - intercept from full model) / intercept from null model, where the null model included all covariates but excluded the main independent variables of interest. Significant interactions (p < .05) were interpreted by calculating the effect of the objective environment variable at −1 SD (i.e., “low”) and +1 SD (i.e., “high”) for continuous variables, and at 0 and 1 for the parks and recreation facilities variables, and for each level of the safety variable (at −1 and +1 SD). The log outcomes were back-transformed for reporting central tendencies and interpreting interaction effects.

## Results

Participant demographic characteristics for each study are presented in Table 2 and descriptive statistics of study variables in Table 3. Final sample size ranged from 1995 to 2068 for NQLS and 687 to 718 for SNQLS due to missing data. NQLS and SNQLS participants indicated similarly low levels of concern about crime safety, with mean scores of 3.3 and 3.4 (range of 1–4), respectively, on the 4- and 5-item scales. Table 4 presents correlations between built environment and safety variables. Tables 5 and 6 show the relation of the environmental and safety variables to minutes/week of physical activity.

**Table 2:** Participant demographic characteristics (NQLS n = 2068, SNQLS n = 718)

	**NQLS**	**SNQLS**
Women	47.6%	53.1%
Non-Hispanic white	73.9%	70.7%
With college degree (or higher)	64.5%	48.8%
Married or living with partner	61.7%	56.8%
Residing in the Baltimore region	40.6%	49.3%
Number of vehicles in household	Mode =1.0	Mode =1.0
	*Mean (SD)*	*Mean (SD)*
Number of vehicles per adult in household	1.04 (0.5)	0.9 (0.5)
Age in years	45.0 (10.9)	74.4 (6.3)
Number of people in household	2.6 (1.4)	1.8 (0.8)
Years at current address	9.6 (9.0)	24.7 (12.5)

SD: Standard Deviation.

**Table 3:** Descriptive statistics for independent variables and outcomes

	**NQLS**		**SNQLS**	
	**Mean (CI or SD)**	**Range**	**Mean (CI or SD)**	**Range**
MVPA min/week (NQLS n = 1995, SNQLS n = 706)	233.0 (225.4–240.2)	0 – 1674.75	93.6 (85.7–102.9)	0 – 825
Walking for transportation min/week (NQLS n = 2065, SNQLS n = 718)	27.3 (24.4–30.5)*	0 – 3360.0	40.9 (35.1–47.3)	0 – 585
Walking for leisure min/week (NQLS n = 2068, SNQLS n = 718)	19.1 (17.1–21.3)*	0 – 3360.0	99.5 (90.9–109.6)	0 – 585
Traffic safety (5 item mean, NQLS n = 2068), (3 item mean, SNQLS n = 717)	2.9 (0.7)	1 – 4	2.7 (0.7)	1 – 4
Pedestrian safety (7 item mean, NQLS n = 2068), (9-item mean SNQLS n =717)	2.9 (0.5)	1.17 – 4	2.7 (0.4)	1.56 – 4
Crime safety (4 item mean, NQLS n = 2067) (5 item mean, SNQLS n = 717)	3.3 (0.6)	1 – 4	3.4 (0.6)	1 – 4
Walkability index (NQLS n = 2068, SNQLS n = 718)	0.0 (3.3)	−5.0 – 13.4	−0.1 (2.8)	−4.1 – 12.5
	**Range %**		**Range %**	
	**Lower dichotomy**	**Upper dichotomy**	**Lower dichotomy**	**Upper dichotomy**
Number parks (NQLS n = 2068, SNQLS n = 718)	0	1.0 – 13.0	0	1.0 – 7.0
	12.5%	87.5%	39.4%	60.6%
Number recreation facilities (NQLS n = 2068, SNQLS n = 718)	0	1.0 – 27.0	0	1.0 – 31.0
	33.4%	66.6%	57.4%	42.6%

CI: Confidence Interval, MVPA: Moderate to Vigorous Physical Activity (measured by accelerometer); SD: Standard Deviation.

*The values reported are exponentiated (back-transformed) from the natural log of these outcomes to improve the distribution for analyses.

**Table 4:** Pearson's correlations between built environment and safety variables

	**Traffic safety**	**Pedestrian safety**	**Crime safety**
NQLS			
Walkability index	.099**	.153**	−.164**
Number parks	.053	.103**	−.135**
Number recreation facilities	.046	.133**	−.127**
SNQLS			
Walkability index	−.070	.184**	−.193**
Number parks	−.046	.204**	−.014
Number recreation facilities	.060	.141**	−.023

**P < .001.

**Table 5:** Unstandardized regression coefficients and P-values for built environment, perceived safety, and their interactions in the NQLS sample

**Outcome variable**									
	**Total MVPA**^**a **^**from accelerometers**			**Natural log of walking for transportation**^**a**^			**Natural log of walking for leisure**^**a**^		
	**n = 1995**			**n = 2065**			**n = 2068**		
	**ICC = .09**^**d**^			**ICC = .04**^**d**^			**ICC = .00**^**d**^		
		**B (95% CI)**	**P-value**		**B (95% CI)**	**P-value**		**B (95% CI)**	**P-value**
Walkability index	M1	9.48 (5.31, 13.65)	.000*	M10	0.18 (0.12–0.25)	.000*			
Traffic safety		17.15 (.04, 24.27)	.049*		0.24 (0.01–0.48)	.042*			
Interaction		−2.63 (−8.22, 2.96)	.357		−0.04 (−0.12–0.03)	.271			
Walkability index	M2	7.81 (3.69, 11.94)	.000*	M11	0.18 (0.12–0.24)	.000*			
Pedestrian safety		40.26 (18.02, 62.51)	.000*		0.46 (0.16–0.76)	.002*			
Interaction		7.48 (.86, 14.09)	.027*		−0.01 (−0.1–0.07)	.796			
Walkability index	M3	9.99 (5.99, 13.99)	.000*	M12	0.18 (0.12–0.24)	.000*			
Crime safety		13.41 (−5.43,32.25)	.163		0.01 (−0.25–0.28)	.925			
Interaction		−6.51 (−12.34, −0.69)	.028*		−.005 (−0.13–0.03)	.199			
Parks	M4	−15.46 (−52.55, 21.63)	.412				M13	−0.03 (−0.48–0.43)	.909
Traffic safety		16.44 (−6.45, 39.33)	.159					0.25 (0.06–0.57)	.112
Interaction		11.11 (−34.42, 56.64)	.632					0.16 (-0.46–0.78)	.618
Parks	M5	−9.70 (−46.98, 27.57)	.609				M14	−0.04 (−0.50–0.43)	.875
Pedestrian safety		17.95 (−11.96, 47.86)	.239					0.39 (−0.02–0.80)	.061
Interaction		76.32 (16.93, 135.71)	.012*					0.17 (−0.64–0.98)	.681
Parks	M6	−1.26 (−41.86, 39.33)	.951				M15	0.22 (−0.30–0.75)	.403
Crime safety		23.02 (−9.72, 55.76)	.168					0.29 (−0.16–0.74)	.205
Interaction		−47.73 (−112.28, 16.82)	.147					−0.83 (−1.71–0.06)	.070
Rec facilities	M7	31.39 (6.10, 56.68)	.015*				M16	0.39 (0.08–0.70)	.013*
Traffic safety		19.38 (1.52, 37.24)	.034					0.22 (−0.01–.046)	.066
Interaction		6.08 (−29.19, 41.35)	.735					0.58 (0.10–1.05)	.018*
Rec facilities	M8	28.10 (2.79, 53.41)	.030*				M17	0.37 (0.04–0.69)	.026*
Pedestrian safety		36.90 (14.07, 59.72)	.002*					0.39 (0.08–0.71)	.013*
Interaction		34.17 (−11.13, 79.47)	.139					0.07 (−0.55–0.69)	.821
Rec facilities	M9	35.66 (9.42, 61.89)	.008*				M18	0.40 (0.06–0.73)	.021*
Crime safety		13.20 (−7.74, 34.19)	.217					−0.05 (−0.33–0.23)	.740
Interaction		−32.32 (−72.61, 7.98)	.116					0.22 (−0.32–0.71)	.421

MVPA = moderate to vigorous physical activity.

B = unstandardized regression coefficient.

CI = confidence interval.

ICC = intraclass correlation coefficient assessing proportion of variance between block groups.

M = model number.

^a^Controlling for age, ethnicity, gender, education, marital status, months at address, number of people in the household and number of vehicles per adult.

^d^ICCs are for the empty model containing no independent variables.

^*^Significant at *p* < 0.05.

**Table 6:** Unstandardized regression coefficients and P-values for built environment, perceived safety, and their interactions in the SNQLS sample

**Outcome variable**									
	**Total MVPA**^**a **^**from accelerometers**			**Walking for transportation**^**a**^			**Walking for leisure**^**a**^		
	**n = 687**			**n = 707**			**n = 709**		
	**ICC = .11**^**b**^			**ICC = .31**^**b**^			**ICC = .04**^**b**^		
		**B (95% CI)**	**P-value**		**B (95% CI)**	**P-value**		**B (95% CI)**	**P-value**
Walkability iIndex	M19	6.03 (2.71, 9.34)	.000*	M28	9.06 (6.69, 11.42)	.000*			
Traffic safety		7.92 (−3.97, 19.82)	.191		−3.80 (−12.20, 4.60)	.375			
Interaction		−3.06 (−7.79, 1.67)	.205		−1.07 (−4.42, 2.27)	.529			
Walkability index	M20	5.57 (2.20, 8.94)	.001*	M29	8.89 (6.51, 11.26)	.000*			
Pedestrian safety		14.14 (−4.42, 32.69)	.082		6.21 (−6.75, 19.17)	.592			
Interaction		0.65 (−6.51, 7.80)	.877		2.44 (−2.56, 7.44)	.634			
Walkability index	M21	6.82 (3.36, 10.28)	.000*	M30	9.46 (6.98, 11.94)	.000*			
Crime safety		14.89 (0.12, 29.66)	.048*		4.31 (−6.11, 14.74)	.417			
Interaction		0.56 (−3.59, 4.71)	.674		0.54 (−2.42, 3.51)	.721			
Parks	M22	33.01 (15.76, 50.26)	.000*				M31	14.52 (−5.14, 34.18)	.147
Traffic safety		6.42 (−5.50, 18.35)	.290					9.13 (−4.76, 23.02)	.197
Interaction		−12.25 (−36.08, 11.58)	.313					−11.65 (−39.31, 16.01)	.409
Parks	M23	31.64 (15.01, 49.79)	.000*				M32	13.13 (−6.68, 32.93)	.203
Pedestrian safety		11.74 (−6.73, 29.84)	.145					16.36 (−4.92, 37.65)	.091
Interaction		5.59 (−28.82, 43.41)	.737					29.85 (−12.38, 72.08)	.131
Parks	M24	33.28 (16.06, 50.50)	.000*				M33	15.18 (−4.36, 34.72)	.127
Crime safety		8.44 (−5.91, 22.79)	.347					7.03 (−9.61, 23.67)	.542
Interaction		1.35 (−25.90, 28.61)	.960					24.38 (−7.27, 56.03)	.140
Rec facilities	M25	10.20 (−7.57, 27.98)	.260				M34	9.22 (−10.71, 29.15)	.364
Traffic safety		7.04 (−5.22, 19.31)	.260					8.32 (−5.88, 22.52)	.251
Interaction		−7.88 (−32.39, 16.63)	.528					−12.02 (−40.44, 16.41)	.407
Rec facilities	M26	7.24 (−10.58, 25.06)	.448				M35	5.05 (−14.73, 24.84)	.632
Pedestrian safety		7.24 (−10.58, 25.06)	.026*					27.51 (6.03, 49.00)	.010*
Interaction		19.65 (−18.40, 57.71)	.309					60.31 (17.30, 103.32)	.014*
Rec facilities	M27	9.97 (−7.79, 27.72)	.268				M36	9.01 (−10.87, 28.89)	.365
Crime safety		9.24 (−5.37, 23.84)	.323					9.39 (−7.45, 26.22)	.364
Interaction		−3.57 (−30.80, 23.66)	.742					11.04 (−20.36, 42.43)	.357

MVPA = moderate to vigorous physical activity.

B = unstandardized regression coefficient.

CI = confidence interval.

ICC = intraclass correlation coefficient assessing proportion of variance between block groups.

M = model number.

^a^Controlling for age, ethnicity, gender, education, marital status, months at address, number of people in the household and number of vehicles per adult.

^b^ICCs are for the empty model containing no independent variables.

^*^Significant at *p* < 0.05.

### NQLS models

#### Total MVPA

**Figure 1:**
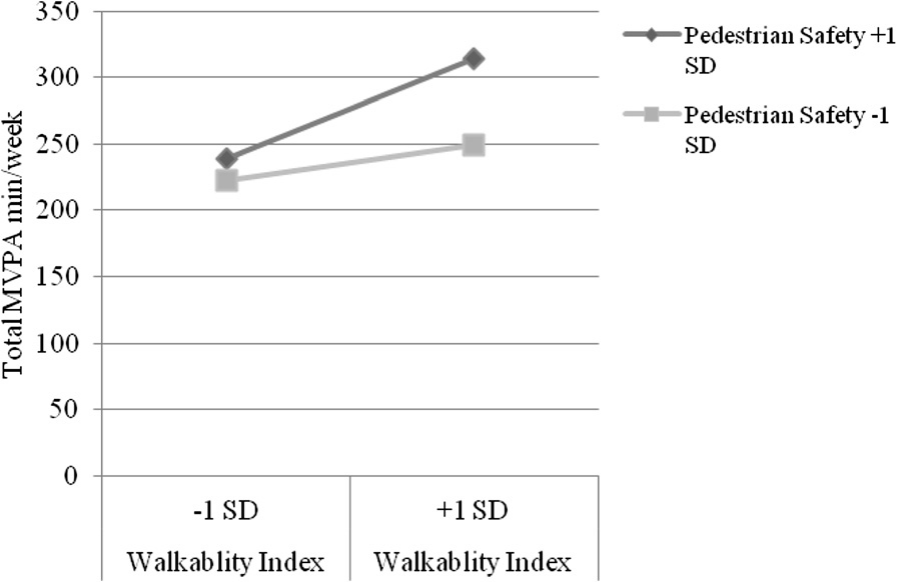
Significant interaction between pedestrian safety and walkability index related to total moderate to vigorous physical activity among younger adults (M2).

**Figure 2:**
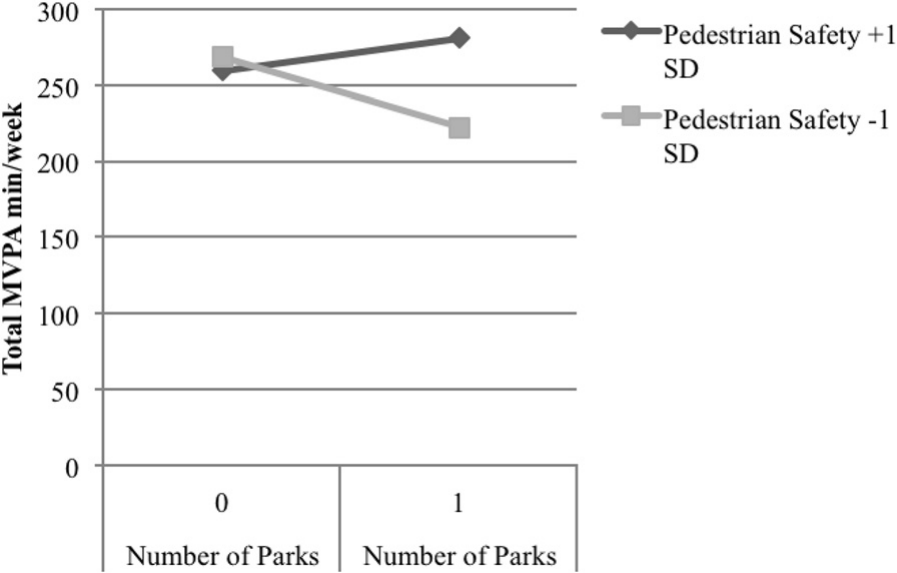
Significant interaction between pedestrian safety and presence of nearby parks related to total moderate to vigorous physical activity among younger adults (M5).

**Figure 3:**
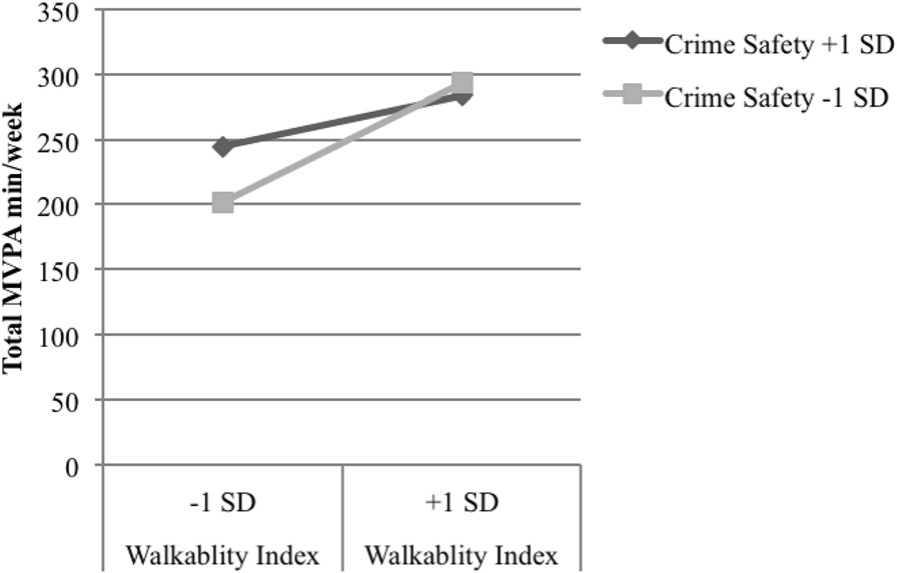
Significant interaction between crime safety and walkability index related to total moderate to vigorous physical activity among younger adults (M3).

NQLS participants engaged in an average of 233.0 minutes/week of total MVPA, as assessed by accelerometer. As shown in Table 5, walkability (see Models 1–3; Bs = 7.81 to 9.99; *p*-values < .001) and number of recreation facilities (see Models 7–9; Bs = 28.10 to 35.66; *p*-values < .05) were consistently associated with total MVPA minutes/week (note that main effects for environmental variables in relation to all three outcomes have been reported previously for the NQLS sample) [31]. Pedestrian safety was associated with total MVPA minutes/week in the model that included the walkability index (see Model 2; B = 40.26; *p* < .001) and the model that included number of recreation facilities nearby (see Model 8; B = 36.90; *p* = .002). Traffic safety was associated with total minutes of MVPA/week in the model that included the walkability index (see Model 1; B = 17.15; *p =* .049). There were positive interactions between walkability and pedestrian safety (see Model 2; B = 7.48; *p =* .027; pseudo R^2^ = .634) and number of parks nearby and pedestrian safety (see Model 5; B = 76.32; *p* = .012; pseudo R^2^ = .103), and a negative interaction between walkability and crime safety (see Model 3; B = −6.51; *p* = .028; pseudo R^2^ = .700) in explaining total MVPA. As shown in Figure 1, for participants with high pedestrian safety, having higher neighborhood walkability accounted for 75.1 additional minutes/week of total MVPA. For participants with low pedestrian safety, having higher neighborhood walkability accounted for 26.4 more minutes/week of total MVPA. As shown in Figure 2, for participants with high pedestrian safety, having 1+ vs. 0 parks nearby accounted for 22.2 additional minutes/week of total MVPA. For participants with low pedestrian safety, having 1+ vs. 0 parks nearby accounted for 46.2 fewer minutes/week of total MVPA. As shown in Figure 3, for participants with high crime safety, having higher walkability accounted for 38.8 additional minutes/week of total MVPA; for participants with low crime safety, having higher walkability accounted for 91.2 additional minutes/week of total MVPA.

#### Walking for transportation

NQLS participants reported an average of 174.1 minutes/week of walking for transportation (not transformed). As shown in Table 5, walkability was consistently associated with (log) minutes/week of walking for transportation (see Models 10–12; Bs = .18; *p*-values < 0.01). Traffic safety was related to (log) walking for transportation in the model that included the walkability index (see Model 10; B = .24; *p =* .042). Pedestrian safety was also related to (log) walking for transportation in the model that included the walkability index (see Model 11; B = .46; *p* = .002). There were no significant interactions.

#### Walking for leisure

**Figure 4:**
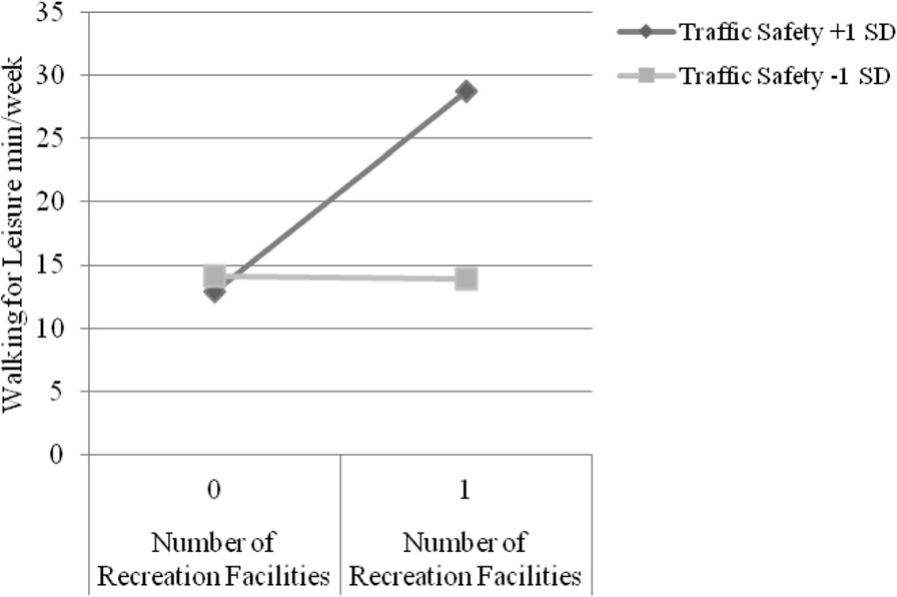
Significant interaction between traffic safety and presence of nearby private recreation facilities related to walking for leisure among younger adults (M16).

NQLS participants reported an average of 115.1 minutes/week of walking for leisure (not transformed). The number of recreation facilities was consistently related to (log) walking for leisure (see Models 16–18; Bs = .37-.40; *p*s *=* .013-.021). Pedestrian safety was related to (log) walking for leisure in the model that included the number of recreation facilities (see Model 17; B = .39; *p* = .013). There was a positive interaction between the number of recreation facilities nearby and traffic safety (see Model 16; B = .58; *p =* .018; pseudo R^2^ = .911). As shown in Figure 4, for participants with high traffic safety, having 1+ vs. 0 recreation facilities nearby accounted for 15.7 additional minutes/week of walking for leisure. For participants with low traffic safety, having 1+ vs. 0 recreation facilities nearby was not related to leisure walking minutes, accounting for 0.2 fewer minutes/week.

### SNQLS models

#### Total MVPA

SNQLS participants engaged in an average of 93.6 minutes/week of accelerometer-based total MVPA. As shown in Table 6, walkability (see Models 19–21; Bs = 5.57 to 6.82) and number of parks (see Models 22–24; Bs = 31.64 to 33.28) were consistently associated with total MVPA minutes/week (*p*-values < 0.01) within the SNQLS sample. Crime safety (see Model 21; B = 14.89; *p* = .048) was associated with total MVPA minutes/week in the model that included the walkability index. Pedestrian safety (see Model 26; B = 19.55; *p* = .026) was associated with total MVPA minutes/week in the model that included the number of recreation facilities nearby. There were no interactions in models explaining total MVPA.

#### Walking for transportation

SNQLS participants reported an average of 40.9 minutes/week of walking for transportation. As shown in Table 6, walkability was consistently associated with minutes/week of walking for transportation (see Models 28–30; Bs = 8.89 to 9.46; *p*-values < 0.01). No safety variables were related to walking for transportation and there were no interactions.

#### Walking for leisure

**Figure 5:**
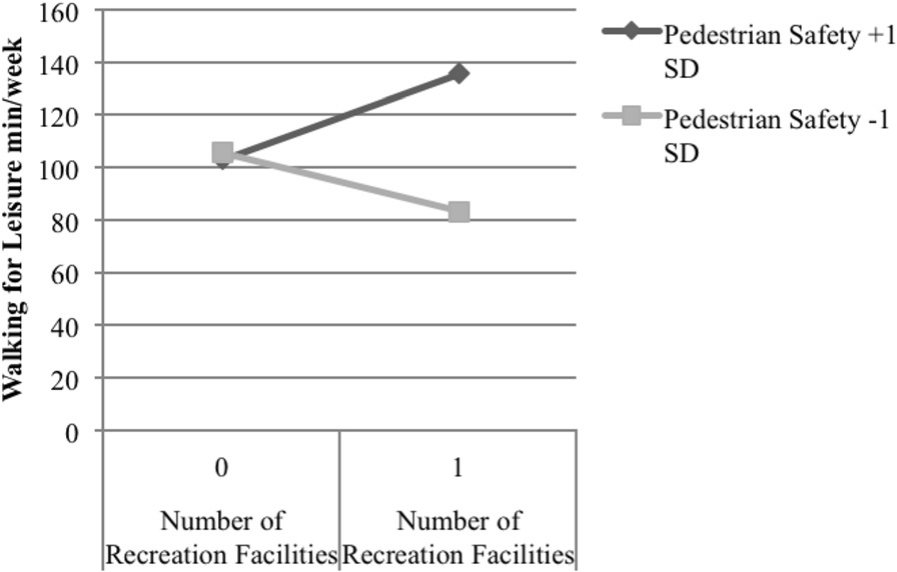
Significant interaction between pedestrian safety and number of nearby private recreation facilities related to walking for leisure among older adults (M35).

SNQLS participants reported an average of 99.5 minutes/week of walking for leisure. As shown in Table 6, pedestrian safety was associated with walking for leisure (see Model 35; B = 27.51; p = .012). There was a positive interaction between number of recreation facilities nearby and pedestrian safety (see Model 35; B = 60.31; p = .014; pseudo R^2^ = .49) in explaining minutes/week of walking for leisure. As shown in Figure 5, for participants with high pedestrian safety, having 1+ recreation facilities nearby accounted for 32.8 additional minutes/week of walking for leisure compared to 0 facilities; for participants with low pedestrian safety, having 1+ recreation facilities nearby accounted for 22.7 fewer minutes/week of walking for leisure compared to those with 0 facilities.

## Discussion

The current study examined interactions between perceived safety and built environment variables in explaining physical activity in samples of younger adults and older adults selected from the same regions and from neighborhoods that varied by walkability and income. The results did not support the expected interaction effects between neighborhood environment and safety variables in either sample, particularly for older adults. There were only 5 significant interactions out of 36 models, and only two of the patterns of association confirmed hypotheses that physical activity would be highest when built environments were favorable and perceived safety was high. The observed pattern of results is interpreted as not supporting perceived safety related to crime, traffic, or pedestrian infrastructure as moderating the relation of built environment variables to physical activity. Thus, the nature of the few significant interactions is not interpreted.

Present findings regarding main effects of perceived safety variables were inconsistent, which has been the case in prior studies. Six of 18 models with the NQLS sample of younger adults had significant safety main effects: 4 involved pedestrian safety and 2 involved traffic safety. Only 3 of 18 models with the SNQLS sample had significant main effects, two involving pedestrian safety and one involving crime safety. The pedestrian safety scale was the most frequently significant as a main effect, as it was related to 5 of the 6 outcomes in at least one model. Across the two age groups, it was related to total MVPA minutes and walking for leisure in several different models. This scale assessed built environment attributes that could protect pedestrians from traffic, such as design of intersections, presence of crosswalks, and presence and quality of sidewalks. The relatively consistent support for pedestrian safety main effects may be because the scale assessed perceptions of specific elements in the built environment (e.g., qualities of street crossings), rather than more subjective concerns about crime or volume of traffic. These positive main effects also suggest that neighborhood pedestrian safety may be one of the more important factors in people’s leisure walking and overall physical activity. Further, pedestrian safety factors can be modified, and the present findings suggest that improving such features can positively impact physical activity and walking. There were only two main effects of traffic safety, so improving measures and testing more complex models may be needed to advance evidence for this variable. The crime scale that dealt with concerns about personal safety had no significant main effect in any of the models. Thus, direct associations between perceived crime safety and physical activity were not supported, consistent with most previous literature [12].

The mixed main effects and null interaction effects suggest that one or more of the following may be true: the current measures of perceived safety (in this case, from the NEWS) lack sensitivity to detect these relationships, the current outcomes (accelerometer-measured MVPA, and self-reported walking for leisure and transportation scales from the IPAQ and CHAMPS ) lack specificity, perceived safety variables are not associated with physical activity, or the links between perceived safety and physical activity are even more complex than could be assessed with these interactions.

The measures of crime, traffic, and pedestrian safety used here may not be sufficiently valid or sensitive to perceptions of safety, warranting better measures. The high mean scores and somewhat small standard deviations suggest a lack of variability and potential ceiling effect, suggesting it may be necessary to develop improved measures or design studies to purposefully select participants with wide variation in perceived safety to ensure hypotheses can be adequately evaluated. The limited variation in safety scores is somewhat surprising because the samples were selected to represent diverse socioeconomic status, and safety variables were documented to differ significantly by neighborhood income [32]. It may also be that the cross-sectional design of the current study is a less sensitive way to uncover these relationships, as opposed to prospective designs. An even better approach would be to conduct quasi-experimental evaluations of efforts to reduce crime, enhance traffic safety, or improve the pedestrian environment.

There are some clues in the criminology literature that could lead to improved measurement and models. First, this literature distinguishes between two related concepts: fear of crime and an assessment of one’s own personal risk of victimization. Fear of crime refers to emotional reactions where perceived risk of victimization is a cognitive judgment of risk [33]. Future research may parse apart these distinctions to assess whether one or both are related to outdoor physical activity behaviors. Further, having witnessed or been the victim of crime can heighten perception of crime [34], so adding such historical variables as a covariate or third variable in the interactions could be informative. Protective strategies to manage perceived crime and traffic safety, such as avoiding “dangerous” places or routes, traveling with a companion, or carrying a cell phone, could also affect associations with physical activity, so future studies could include such variables in analyses.

The physical activity outcomes examined in this study may lack sufficient specificity to illuminate connections to perceived safety. The amount of physical activity that participants reported on the IPAQ (NQLS) or CHAMPS (SNQLS) or that was demonstrated via use of accelerometers did not necessarily occur in the participants’ neighborhoods. There was a mismatch in locations between the non-specific physical activity measures and neighborhood-specific NEWS safety items. Location-specific physical activity outcomes may help to elucidate these relationships.

Research has pointed to the potential negative effects of safety perceptions on physical activity, particularly for older adults who tend to be more fearful and less active overall [12,35]. However, both the sample of younger adults and the sample of older adults in the present study had similarly high perceptions of safety from crime and related personal dangers, indicating that crime safety may not be as much of a driving factor in older adults’ low levels of physical activity as has been proposed [36,37].

This was one of the first studies to use an ecological model to examine built environment by safety interactions in explaining physical activity in seniors and younger adults [38]. The present study employed parallel analyses across two large samples using similar methods, which were also strengths. The weaknesses of the present study were that it relied on cross-sectional data, limiting the conclusions that can be drawn, and that the results have limited generalizability due to sampling bias (high percentages in both samples were white and college-educated). Though the age groupings used in the present study differentiating the younger sample from the older sample are fairly standard, the younger adult sample had a very wide age range (20–65 years), so it is possible there are age-related differences within the younger adult sample that were not revealed in present analyses. The IPAQ has been shown to be valid for total MVPA, but validity of component scores, such as walking, has not been demonstrated [27]. The same is true of the CHAMPS. It is not known from these data where the participants’ MVPA, leisure, or transportation walking actually took place. To the extent the activities occurred outside of the neighborhood, any impact of the safety variables would likely be obscured. The analytic approach of separate models for each interaction was based on a desire to be as sensitive as possible to detecting significant interactions, but this method raises the likelihood of type 1 error. Given that the findings were generally null, type 1 error was not a problem.

## Conclusion

Few main effects of perceived safety factors or their interactions with objective built environment factors were significant correlates of objectively measured total physical activity or self-reported walking for leisure or transportation across samples of younger and older adults. The null results are generally consistent with prior studies [39-41]. The proposed hypotheses about crime, traffic, and pedestrian safety warrant future exploration with more refined methods and interactions with different variables. Location-specific physical activity outcomes may prove to be more informative. Future research should draw on insights from criminological research on worry about crime and constrained behaviors, which suggests there may be other factors influencing and moderating these relationships, such as the use of protective strategies, having been a victim of a crime, witness to a crime, or having knowledge of someone being victimized [34]. Associations between safety perceptions and physical activity may vary by sex and socioeconomic status, so these interactions should also be examined. We encourage continued exploration of multiple safety domains, including improved measurement approaches as well as consideration of personal crime- or injury-related histories. Quasi-experimental studies of interventions to improve crime, traffic safety, and pedestrian safety infrastructure on physical activity may also be instructive [37].

## Abbreviations

CHAMPS: Community Healthy Activities Model Program for Seniors; IPAQ: International Physical Activity Questionnaire; NEWS: Neighborhood Environment Walkability Scale; NQLS: Neighborhood Quality of Life Study; MVPA: Moderate to Vigorous Physical Activity; SNQLS: Senior Neighborhood Quality of Life Study.

## Competing interests

Sallis is stockholder and on the Board of Directors of Santech Inc. The other authors have no competing interests to disclose.

## Authors’ contributions

NLB conceptualized and drafted the current manuscript, analyzed and interpreted data. RAM analyzed and interpreted data and drafted sections of this manuscript. JAC analyzed and interpreted data and drafted sections of this manuscript. JFS was the primary investigator of NQLS and Co-PI of SNQLS. He contributed to the conceptualization of the study questions, drafted sections of the manuscript, and offered revisions on the entire manuscript. KLC directed the data collection on both SNQLS and NQLS studies, compiled and analyzed the accelerometer data reported here. TLC was co-investigator on both SNQLS and NQLS studies; she prepared both data sets for analysis, helped to design the analysis plan for this manuscript, and offered feedback on drafts of the manuscript. BES was a key investigator on the NQLS study who contributed substantially to the conception and design of the study of younger adults and revised drafts of the current manuscript. LDF was a key investigator on both SNQLS and NQLS studies who led the GIS component of these studies, including analysis and interpretation of these data; he edited the manuscript. ACK was PI of SNQLS study and contributed substantially to the conception and design of the study of older adults and revised drafts of the current manuscript. JK was a key investigator on both SNQLS and NQLS studies who contributed substantially to the conception and design of both studies and revised drafts of the current manuscript. All authors gave their final approval of the submitted version.
